# Proteomic Profiling of Extracellular Vesicles Released by Leptin-Treated Breast Cancer Cells: A Potential Role in Cancer Metabolism

**DOI:** 10.3390/ijms232112941

**Published:** 2022-10-26

**Authors:** Luca Gelsomino, Ines Barone, Amanda Caruso, Francesca Giordano, Matteo Brindisi, Giovanna Morello, Felice Maria Accattatis, Salvatore Panza, Anna Rita Cappello, Daniela Bonofiglio, Sebastiano Andò, Stefania Catalano, Cinzia Giordano

**Affiliations:** 1 Department of Pharmacy, Health and Nutritional Sciences, Via P. Bucci, University of Calabria, Arcavacata di Rende (CS), 87036 Cosenza, Italy; 2 Centro Sanitario, Via P. Bucci, University of Calabria, Arcavacata di Rende (CS), 87036 Cosenza, Italy; 3 Cell Adhesion Unit, San Raffaele Vita-Salute University, 20132 Milan, Italy; 4 Institute for Biomedical Research and Innovation, National Research Council, 95121 Catania, Italy

**Keywords:** breast cancer, extracellular vesicles, leptin, macrophages, metabolism

## Abstract

Tumor extracellular vesicles (EVs), as endocytic vesicles able to transport nucleic acids, proteins, and metabolites in recipient cells, have been recognized fundamental mediators of cell-to-cell communication in breast cancer. The biogenesis and release of EVs are highly regulated processes and both the quantity of EVs and their molecular cargo might reflect the metabolic state of the producing cells. We recently demonstrated that the adipokine leptin, whose circulating levels correlate with adipose tissue expansion, is an inducer of EV release from breast cancer cells. Here, we show a specific proteomic signature of EVs released by MCF-7 breast cancer cells grown in the presence of leptin (Lep-EVs), in attempt to find additional molecular effectors linking obesity to breast cancer biology. An analysis of the proteomic profile of Lep-EVs by LC-MS/MS revealed a significant enrichment in biological processes, molecular functions, and cellular components mainly related to mitochondrial machineries and activity, compared to protein content of EVs from untreated breast cancer cells. Metabolic investigations, carried out to assess the autocrine effects of these vesicles on breast cancer cells, revealed that Lep-EVs were able to increase ATP levels in breast cancer cells. This result is associated with increased mitochondrial respiration evaluated by Seahorse analyzer, supporting the concept that Lep-EVs can modulate MCF-7 breast cancer cell oxidative metabolism. Moreover, taking into account the relevance of tumor immune cell crosstalk in the tumor microenvironment (TME), we analyzed the impact of these vesicles on macrophage polarization, the most abundant immune component in the breast TME. We found that tumor-derived Lep-EVs sustain the polarization of M0 macrophages, derived from the human THP-1 monocytic cells, into M2-like tumor-associated macrophages, in terms of metabolic features, phagocytic activity, and increased expression of CD206-positive population. Overall, our results indicate that leptin by inducing the release of EV-enriched in mitochondrial proteins may control the metabolism of MCF-7 breast cancer cells as well as that of macrophages. Characterization of tumor-derived EV protein cargo in an obesity-associated milieu, such as in the presence of elevated leptin levels, might allow identifying unique features and specific metabolic mechanisms useful to develop novel therapeutic approaches for treatment of breast cancer, especially in obese patients.

## 1. Introduction

The adipocyte-secreted factor leptin, first discovered as an important regulator of appetite and energy balance homeostasis, is now recognized as one of the most important adipokine influencing mammary gland physiopathology. In particular, many epidemiological and experimental observations have identified leptin, whose serum levels increase proportionally to adipose tissue mass, as a key actor for the molecular connection between obesity and breast cancer [1,2]. Expression of leptin and its receptor in breast cancer samples correlates with tumor aggressiveness [3,4], and leptin/leptin receptor signaling activation is involved in breast cancer progression and metastasis [1,5,6]. Serum leptin levels were found to be significantly higher in postmenopausal estrogen receptor-positive breast cancer patients with advanced tumor stage and distant metastases [7]. In addition, body mass index (BMI) and leptin resulted correlated with pathological tumor classification (pT), tumor size, nodal and metastasis status (TNM) in postmenopausal breast cancer patients [8]. Moreover, in obese breast cancer patients, leptin levels were significantly increased in the tumor microenvironment compared to circulating plasma samples [9], suggesting an important role for this adipokine as an active player in tumor microenvironment communication. Indeed, leptin is able to shape the tumor microenvironment, through its ability to induce migration of endothelial cells and to sustain the recruitment of macrophages and monocytes, thus exerting pro-inflammatory effects [6,10,11]. Recently we also demonstrated that this adipokine regulates the release of the small tumor-derived extracellular vesicles (EVs) by epithelial mammary carcinoma cells [12], further highlighting its involvement in cell-to-cell communication in breast cancer.

Small EVs are nano-sized membrane-derived vesicles containing a plethora of bioactive molecules (i.e., proteins, nucleic acid, lipids, and metabolites) that reflects the molecular profile of cells of origin. Tumor-derived EVs by transferring their cargo to recipient cells promote a wide range of cellular processes involved in breast cancer progression [13], extracellular matrix remodeling [14], epithelial-to-mesenchymal transition [15], immune evasion [16,17,18], endocrine- and target-therapy resistance [19,20,21,22] and premetastatic niche formation [23,24]. In recent decades, many efforts have been made to decipher the molecular cargo of tumor-derived EVs along with its modification during disease progression or in relation to the different environmental stimuli, in order to identify novel putative biomarkers to improve non-invasive liquid biopsy tools for cancer detection, classification and tailored therapeutic interventions. For instance, an elegant paper by Hoshino et al. reported that the proteomic analysis of plasma-derived EV across multiple cancer types, including breast carcinoma, identifies the cancer-associated proteomic signature [25]. In addition, it was demonstrated that the proteomic profile of EVs from nine different breast cancer cell lines closely reflects the associated clinical pathophysiology of different breast cancer subtypes [26].

In this study, we focused on the identification of a specific leptin-induced EV proteomic signature in MCF-7 breast cancer cells, and their possible impact on tumor biology, in attempt to find molecular effectors associated with breast cancer progression. Characterization of tumor-derived EV protein cargo in an obesity-associated milieu, such as in the presence of elevated leptin levels, might allow identifying unique features and specific biological processes to be targeted in the clinical management of breast cancer.

## 2. Results

### 2.1. Extracellular Vesicles from Leptin-Treated MCF-7 Breast Cancer Cells Show an Enrichment in Proteins Involved in Energetic Metabolism 

**Figure 1:**
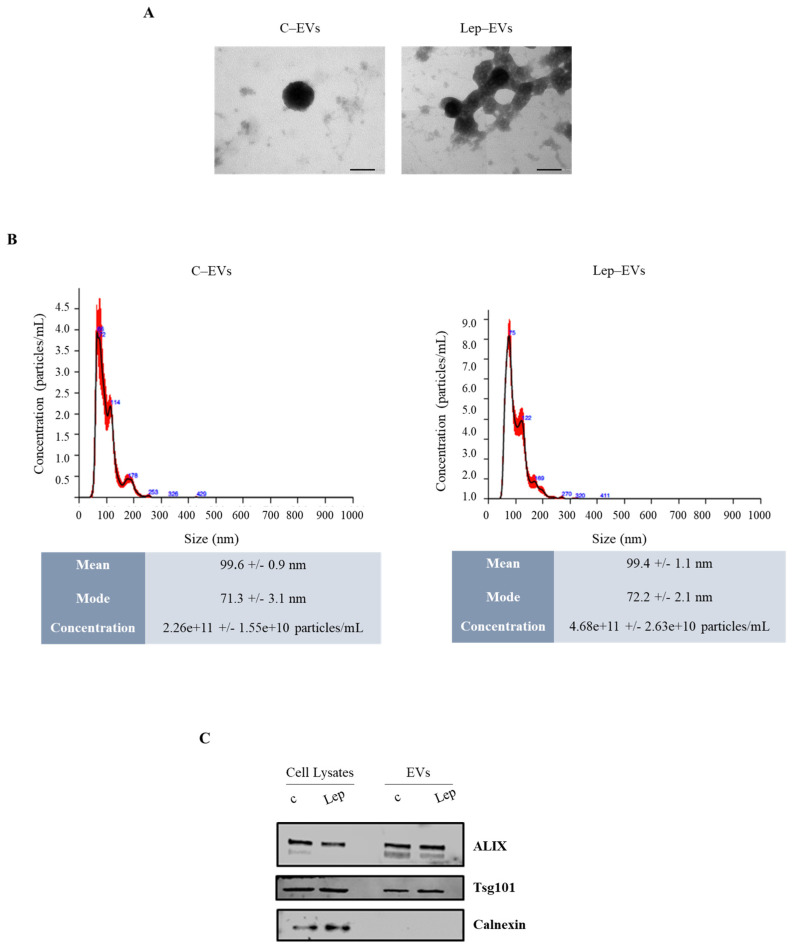
Characterization and quantification of extracellular vesicles (EVs) from MCF-7 breast cancer cells. EVs were isolated from conditioned media of MCF-7 after treatment with vehicle (**c**) or leptin (Lep, 500 ng/mL) for 48 h, and named C-EVs and Lep-EVs, respectively. (**A**) Representative images of transmission electron microscopy showing fields of C-EVs and Lep-EVs. Scale bar 50 nm. (**B**) Representative size distribution profiles of C-EVs and Lep-EVs, measured by nanoparticle tracking analysis. (**C**) Immunoblot analysis showing expression of the EV hallmarks Alix and Tsg101, in equal amount of whole cell lysates and EV lysates of MCF-7 cells as reported. Calnexin was used to ensure that EV samples were not contaminated with endoplasmic reticulum proteins.

First, using sequential ultracentrifugation method [27] we isolated extracellular vesicles (EVs) from conditioned media (CM) of ER-α positive MCF-7 breast cancer cells grown (Lep-EVs) or not (C-EVs) in the presence of leptin (500 ng/mL) for 48 h as previously described [12]. Purified particles were fully characterized using Transmission Electron Microscopy (TEM), Nanoparticle Tracking Analysis (NTA) (Figure 1A and B, respectively), and by evaluating protein expression of selective EV biomarkers (Figure 1C). As expected, particle count revealed an increased concentration of EVs in CM from leptin-treated cells [12] (Figure 1B). 

**Figure 2:**
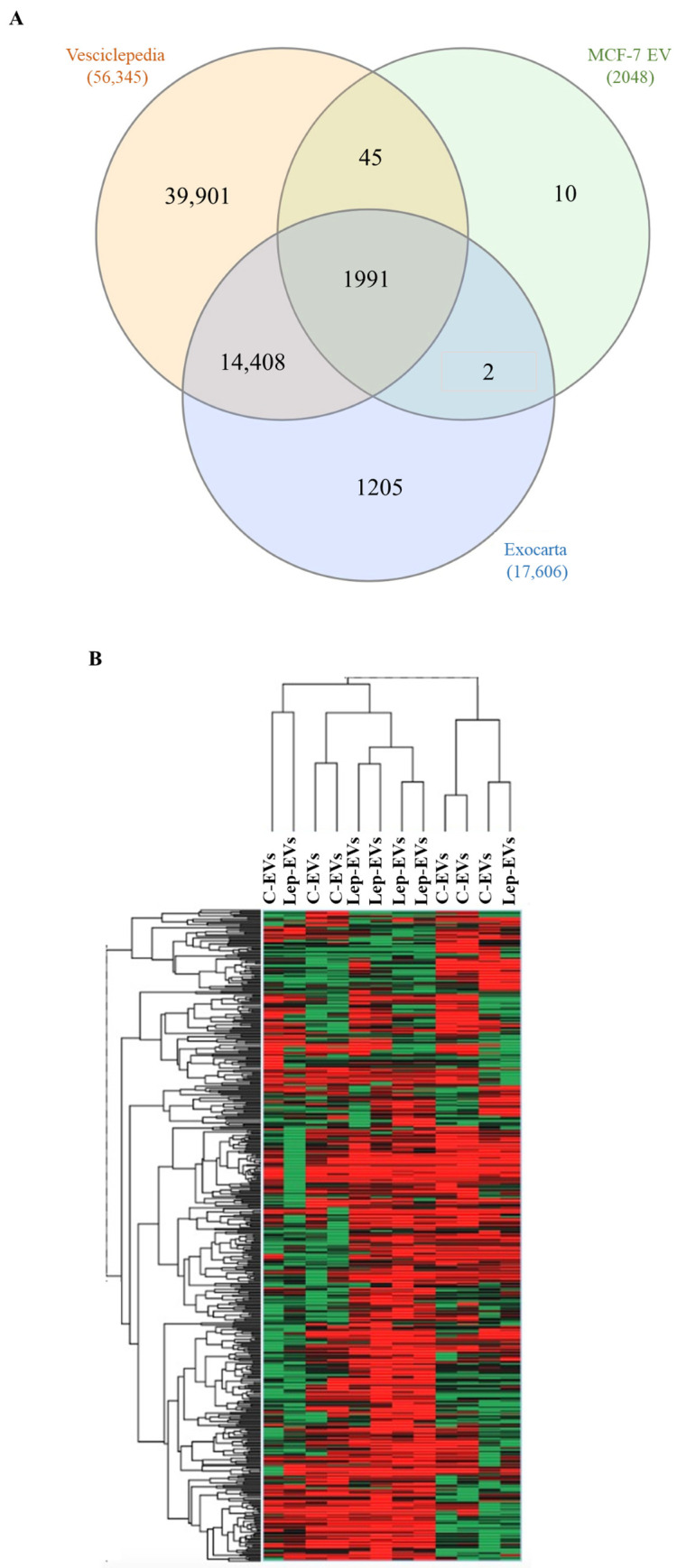
Proteomic profiling of EV lysates from breast cancer cells. (**A**) Venn diagram of proteins identified in the EVs from MCF-7 breast cancer cells compared with proteins annotated in the Vesiclepedia and Exocarta databases using Bioinformatics & Evolutionary Genomics (https://bioinformatics.psb.ugent.be/webtools/Venn/, accessed on 1 November 2021) tool. (**B**) Heatmap of the differentially expressed proteins in C-EVs and Lep-EVs identified by LC-MS/MS analysis. The color scale illustrates the relative expression of each protein across the 12 samples. Heatmap coding uses increasing brightness of red for degree of up-regulation and green for down-regulation. Black color stands for a median expression level.

Next, we analyzed the protein content of C-EVs and Lep-EVs by label-free liquid chromatography-tandem mass spectrometry analysis (LC-MS/MS). A total of 2048 proteins were identified with at least two unique peptides. The list of identified protein analyzed using Bioinformatics & Evolutionary Genomics (https://bioinformatics.psb.ugent.be/webtools/Venn/, accessed on 1 November 2021) against publicly available database Vesiclepedia (www.microvesicles.org, accessed on 1 November 2021) and ExoCarta (http://www.exocarta.org/, accessed on 1 November 2021) for vesicle proteins allow obtaining a Venn diagram (Figure 2A) showing that the majority of the proteins identified in the MCF-7-derived particles are of vesicular origin. Moreover, referring to the top 100 proteins associated with extracellular vesicles in the Vesiclepedia database analysis, 39 proteins were identified in the proteomic profiles of our samples. Among traditional extracellular vesicle markers, we found CD9, CD63, CD81, FLOT1, FLOT2 and different members of the RAB family proteins, which confirm the vesicle origin of analyzed proteins. Although we found elevated biological variability in the protein cargo detected by LC-MS/MS due to the nature of the sample [28], the unsupervised hierarchical clustering analysis of the proteins differentially expressed clustered the protein content of Lep-EVs and C-EVs MCF-7 cells (Figure 2B). 

**Figure 3:**
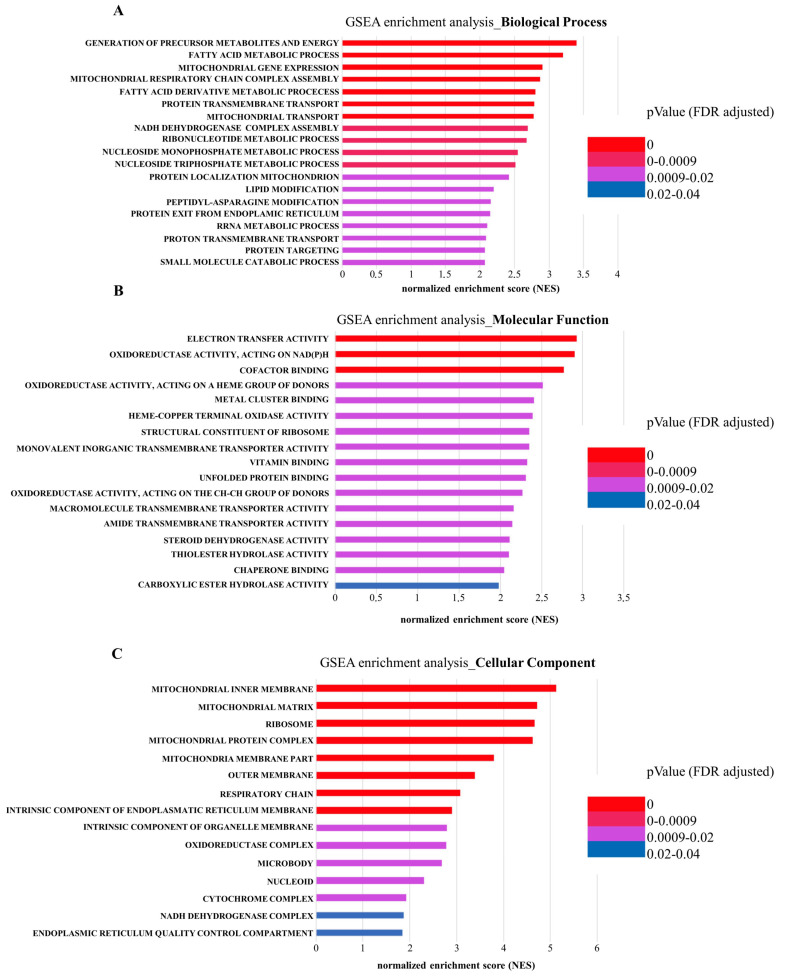
GSEA enrichment analysis of the differentially up-regulated proteins Lep-EVs vs. C-EVs. GO enriched Biological Process (**A**) Molecular Function (**B**) and Cellular Component (**C**) were shown. X-axis: normalized enrichment score (NES) and *p*-value were reported.

Quantitative proteomic analysis revealed extensive changes in the protein cargo of Lep-EVs. Thus, to gain functional insight into the differential proteomic cargo of these EVs released from leptin-treated cells, we performed Gene Ontology (GO) analysis using GSEA (Gene set enrichment analysis). Specifically, to obtain GO enrichments in biological processes, molecular functions, and cellular components we used the entire list of the up-regulated proteins. As shown in Figure 3A–C, we identified several significantly enriched biological process, molecular function, and cellular components mainly related to mitochondrial activity. These data allow us to hypothesize that EVs released from breast cancer cells grown in the presence of leptin may influence metabolic phenotype in recipient cells by transferring the energy producing machinery.

### 2.2. Extracellular Vesicles Released by Leptin-Treated MCF-7 Cells Sustain Mitochondrial Metabolism in Breast Cancer Cells

**Figure 4:**
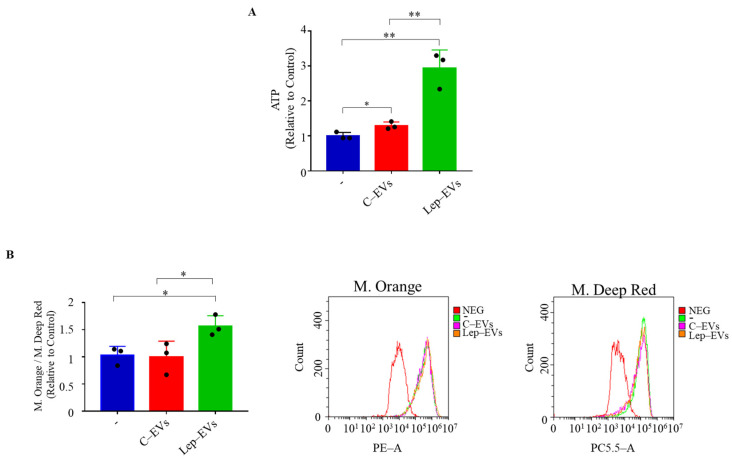
Effects of Lep-EVs on breast cancer cell energy production and on mitochondrial function. (**A**) ATP profiling production in MCF-7 breast cancer cells untreated (–) or treated with C-EVs and Lep-EVs (2 μg/mL) for 48 h. (**B**) Mitochondrial membrane potential and mitochondrial mass were assessed using MitoTracker Orange CM-H2TMRos and MitoTracker Deep Red probes, respectively, in MCF-7 breast cancer cells untreated (–) or treated with C-EVs and Lep-EVs (2 μg/mL) for 48 h. The ratio between mitochondrial membrane potential and mitochondrial mass and representative histograms of mean fluorescence intensity are reported. Each circle represents one independent experiment. Values are expressed as mean ± SD of three independent experiments each performed in triplicates. * *p* ≤ 0.05; ** *p* ≤ 0.01.

On the basis of the above-mentioned results, we investigated the potential ability of the pure extracellular vesicles released by leptin-treated (Lep-EVs) or untreated (C-EVs) MCF-7 cells in remodeling cell metabolism analyzing the ATP levels and the mitochondrial number and function. As shown in Figure 4A, we observed that Lep-EVs significantly enhance the amount of ATP produced in MCF-7 cells compared to the ATP levels found in cells treated with C-EVs. Moreover, in the same experimental conditions we assessed both mitochondrial membrane potential and mitochondrial mass using MitoTracker Orange and Deep Red, respectively. Results revealed that the ratio of mitochondrial membrane potential/mitochondrial mass, representing an index of functionality per mitochondrion, was significantly increased in cells treated with Lep-EVs compared to C-EVs (Figure 4B). Thus, Lep-EVs in breast cancer cells could shape the energy production by altering mitochondrial function.

**Figure 5:**
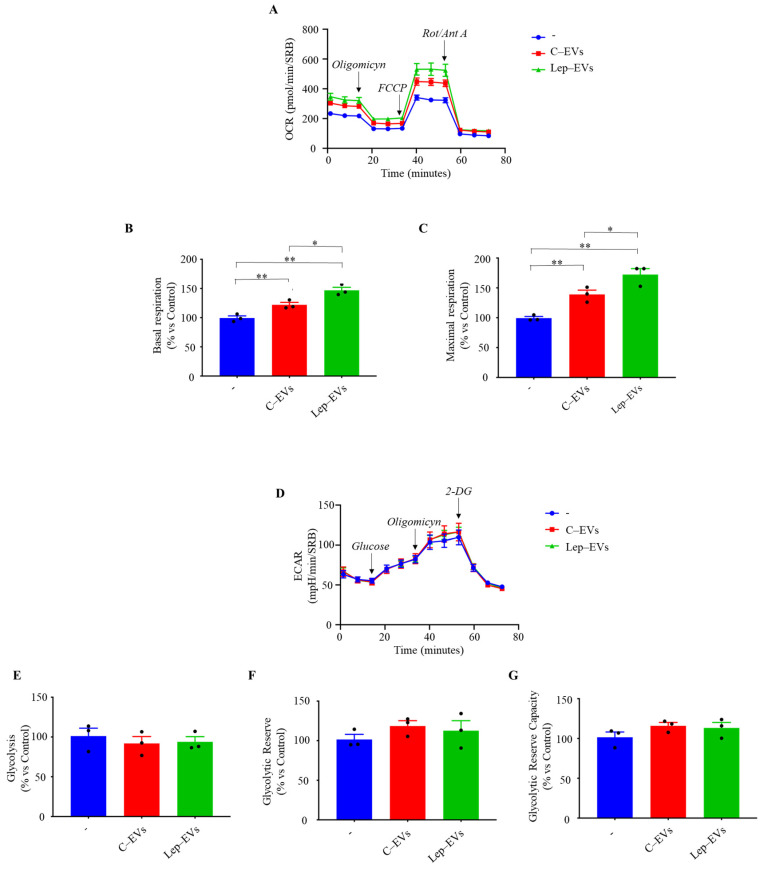
Lep-EVs sustain mitochondrial metabolism of MCF-7 breast cancer cells. The mitochondrial and glycolytic metabolic profile of MCF-7 cells untreated (–) or treated with C-EVs and Lep-EVs (2 μg/mL) for 48 h were assessed using the Seahorse XF-e96 analyzer. Schematic tracing of OCR flux (**A**) and relative histograms of different mitochondrial respiration parameters such as basal (**B**) and maximal (**C**) respiration were reported. (**D**) ECAR representative tracing and relative histograms of glycolysis (**E**), glycolytic reserve (**F**), and glycolytic reserve capacity (**G**) were reported. Each circle represents one independent experiment. Data represent the mean ± SEM of five biological replicates of three independent experiments and normalized by protein content (SRB assay). * *p* ≤ 0.05; ** *p* ≤ 0.01.

To corroborate these findings, we analyzed the two major energy producing pathways, mitochondrial respiration and glycolysis, using the Seahorse Extracellular Flux (XFe96) Analyzer, which allows using the real-time measurements of the Oxygen Consumption Rate (OCR) and Extracellular Acidification Rate (ECAR). Specifically, OCR is a marker for mitochondrial OXPHOS and ATP production, while ECAR is a measure of glycolytic flux. To evaluate the effects of Lep-EVs on the different parameters of cellular respiration, the Mito Stress test was performed on MCF-7 cells, after 48 h of treatment of equal amounts of C-EVs and Lep-EVs, by monitoring the change in OCR following the addition of Oligomycin, FCCP and Rotenone/Antimycin A. As shown in Figure 5A, Lep-EVs increased cellular bioenergetics in MCF-7 cells as measured by OCR, compared to C-EVs. Quantification of the results shows a significant increase in the basal and maximal respiration in breast cancer cells treated with Lep-EVs compared to untreated cells, but also compared to cells treated with EV from control condition (Figure 5B,C). In addition, the Glycolysis stress test was performed by monitoring the ECAR variations following the consecutive additions of Glucose, Oligomycin and 2-deoxy-glucose (2-DG). We found that the basal ECAR remained unchanged in cells treated for 48 h with both C- and Lep-EVs (Figure 5D) along with the glycolytic profile in terms of glycolytic rate, glycolytic reserve, and glycolytic reserve capacity (Figure 5E–G). 

Moreover, we evaluate the direct effect of the adipokine leptin in our cell model to prove the specificity of EV effect on MCF-7 breast cancer cell metabolism. As expected, leptin exposure in MCF-7 breast cancer cells induced a significant increase in OCR, basal respiration, and maximal respiration to a larger extent compared to the effects of pure Lep-EVs. On the other hand, ECAR, glycolysis, glycolytic reserve, and glycolytic reserve capacity resulted in significantly increased leptin-treated cells, but not in Lep-EV-treated cells (Figure S1). These results underline that EV released by leptin-treated breast cancer cells may contribute to the specific increase in mitochondrial OXPHOS metabolism in an autocrine manner.

### 2.3. Extracellular Vesicles from Leptin-Treated MCF-7 Cells Influence Metabolic Profile of Macrophages

**Figure 6:**
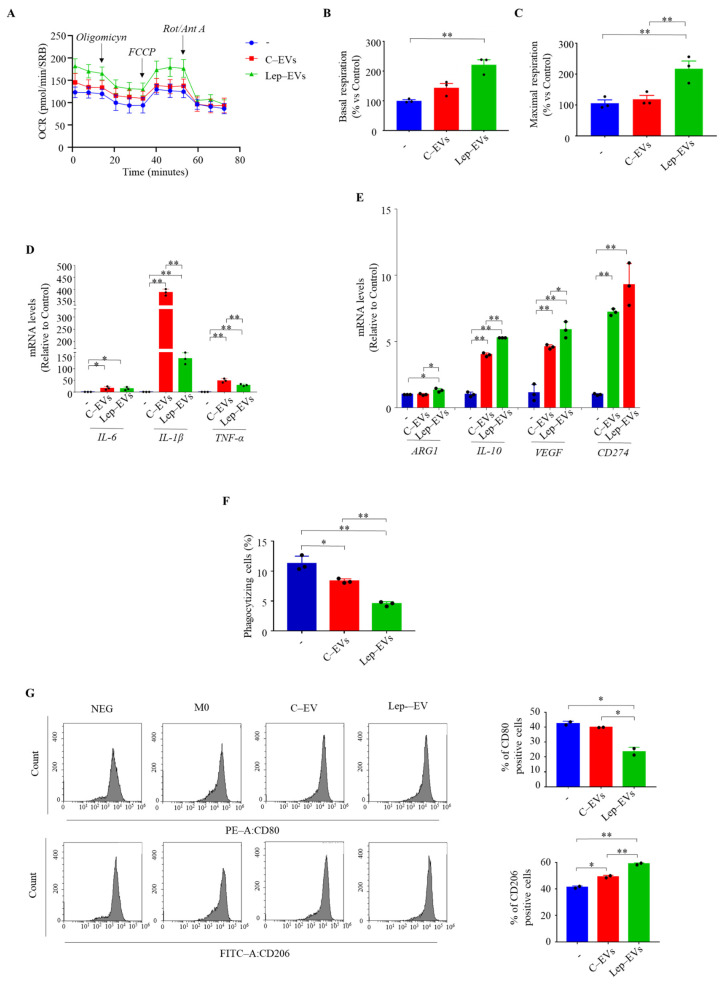
Lep-EVs support the M2-like pro-tumoral macrophage phenotype. THP-1 cells were stimulated with phorbol 12-myristate 13-acetate (PMA, 100 nM) for 24 h followed by 24 h rest to obtain THP-1 macrophage-like cells (M0). The mitochondrial metabolic profile of M0 macrophages untreated (–) or treated with C-EVs and Lep-EVs (2 μg/mL) for 48 h was assessed using the Seahorse Xfe-96 analyzer. A schematic tracing of OCR flux (**A**) and graphic representation of the basal respiration (**B**) and maximal respiration (**C**) were reported. Data represent the mean ± SEM of five biological replicates of three independent experiments and normalized by protein content (SRB assay). (**D**,**E**). Real-time RT-PCR assay for interleukin (IL)-6, *IL-1β*, Tumor Necrosis Factor alpha (*TNF-α*), arginase-1 (*ARG1*), *IL-10*, Vascular Endothelial Factor (*VEGF*), cluster of differentiation 274 (*CD274*), in M0 untreated (–) or treated with C-EVs and Lep-EVs (2 μg/mL) for 48 h. (**F**) M0 macrophages treated as above were incubated with latex beads conjugated with FITC-IgG for 2 h and results were expressed as percentage of phagocytizing cells. (**G**) Flow cytometry analyses of M1 marker CD80 and M2 marker CD206 in M0 macrophages untreated or treated with C-EVs and Lep-EVs for 48 h. The bars represent the percentages of positive cells. Each circle represents one independent experiment. The values represent the mean ± SD of two/three different experiments, each performed in triplicate. * *p* ≤ 0.05; ** *p* ≤ 0.01.

It is well known that metabolic communication between tumor cells and stromal components into the tumor microenvironment fuels tumor progression and might affect therapeutic response sustaining a defective tumor immune surveillance [29]. Thus, we wondered if the message carried by Lep-EVs could mediate the metabolic interaction between breast cancer cells and macrophages, the most important immune cells in the breast tumor microenvironment. To this end, we evaluated changes in metabolic profile of human THP-1 monocytic cells differentiated into M0 macrophages after 48 h of growth in the presence of C-EVs or Lep-EVs. First, we assessed the metabolic profile of macrophages polarized from THP-1 cells as previously described [30]. As expected [31], M1-like macrophages obtained after LPS treatment showed a slight decrease in OCR compared to M0 cells, while the M2-like macrophages obtained after IL-4 treatment revealed a significant increase in OCR, as well as basal respiration associated with an increased oxidative potential (data not shown). Interestingly, we found that M0 macrophages after 48 h of treatment with Lep-EVs displayed a significant increase in OCR, in basal respiration and maximal respiration, compared to untreated M0, while this effect was no evident in M0 grown in the presence of C-EVs (Figure 6A–C). 

In the same experimental conditions, no changes were revealed in the ECAR profile analyzed by the Glycolysis stress test (Figure S2). 

These results suggest that Lep-EVs might support the pro-tumoral macrophage M2-like phenotype generally found in tumor-associated macrophages (TAMs), although the anti-tumor/pro-inflammatory M1-like and pro-tumor M2-like TAMs coexist within tumors [32,33]. Thus, we investigated the potential effects of EVs on modulating the mRNA expression of several markers associated with M1- and M2-like TAMs in M0 polarized macrophages. As revealed by qRT-PCR C-EVs increased M1-like TAM markers such as *IL-6*, *IL-1β* and Tumor Necrosis Factor alpha (*TNF-α*), in a higher extent compared to Lep-EVs (Figure 6D). Moreover, M2-like TAM markers such as arginase-1 (*ARG-1*), *IL-10*, vascular endothelial growth factor (*VEGF*) and cluster of differentiation 274 (*CD274*, also known as Programmed cell death 1 ligand, PD-L1) were found to be up-regulated after treatment with both EVs, but these genes were significantly enhanced in Lep-EV treated M0 polarized macrophages (Figure 6E). In the same experimental conditions, we assessed the phagocytic activity in M0 polarized macrophages. As shown in Figure 6F, cells treated with Lep-EVs displayed significantly reduced phagocytic activity compared to cells receiving the C-EVs. Moreover, we evaluate the polarization state of M0 macrophages untreated or treated with C-EVs or Lep-EVs by flow cytometry analyses for the expression of CD80 and CD206 the well-known markers of M1-like and M2-like cells, respectively. As shown in Figure 6G, treatment with Lep-EVs significantly increased the percentage of CD206 positive cells, while reducing the percentage of CD80 positive cells. 

These data support the hypothesis that EVs released from breast cancer cells grown in the presence of leptin were able to shape macrophages toward to M2-like TAM phenotype. 

## 3. Discussion

Metabolic plasticity is one of the hallmarks of cancer through which tumor cells and tumor microenvironment components constantly rewire their metabolism to satisfy the energetic demands for growth and progression of disease. Emerging findings attribute an important role to extracellular vesicles, mainly small EVs, in mediating the metabolic reprogramming in cancer [34], although the underlying regulatory mechanisms have not been completely deciphered. Here, we provide evidence that the adipokine leptin influences the protein cargo and the biological activities of EVs secreted from breast cancer cells, affecting in both autocrine and paracrine manners the metabolism of breast cancer cell itself and of macrophages, the most abundant immunological component in the tumor microenvironment. For a long time, it has been accepted that in cancer cells, the mitochondrial respiration is impaired, and cells exploit the aerobic glycolysis for glucose metabolism, a mechanism know as Warburg’s effect [35]. Now it is clear that a huge heterogeneity exists in the metabolic phenotype of cancer cells that enables cells to produce ATP while preserving anabolic functions necessary for their survival and proliferation [36]. Several findings revealed the multifaceted direct effects of the obesity hormone leptin in cancer cell metabolism [37]. Park and colleagues [38] demonstrated that leptin receptor signaling affects breast cancer cell metabolism by suppressing mitochondrial respiration in animal models, and enhancing the classically described Warburg’s effect. However, the role of leptin in regulating breast cancer cell proliferation has also been associated with a shift in ATP production from glycolysis to mitochondria and with an improvement in mitochondrial quality, function and biogenesis [39]. In vitro studies revealed that leptin sustained mitochondrial respiration to produce ATP by fostering the use of fatty acids as a fuel for producing energy in cancer cells [40]. More recently, Liu et al. reported that leptin induced the ATP production, in estrogen receptor-positive breast cancer cells, by regulating fatty acid oxidation and oxidative phosphorylation (OXPHOS) via c-Myc/PGC-1 pathway [41]. In line with this evidence, our current findings revealed that in breast cancer cells, the adipokine leptin might influence tumor metabolism, by inducing the release of EVs enriched in molecular component of mitochondrial energetic machinery. Gene set enrichment analysis (GSEA) revealed that Lep-EVs were strongly enriched in gene sets linked to biological process and molecular function related to mitochondrial structure and activity. The possibility to find mitochondrial material within EVs has been largely reported particularly in neurodegenerative diseases [42]. In addition, the self-protective mitochondrial transfer has been described in many cancer types, such as multiple myeloma [43], melanoma [44], leukemia [45,46], lung [47], prostate [48], and breast cancer [49,50,51,52], and it appears to be an important biological mechanism in cancer able to sustain energetic metabolism, tumorigenesis, and therapy resistance [53]. The described molecular mechanisms of mitochondrial transfer indicate that tunneling nanotubes and larger extracellular microvesicles formed by blebbing of the cellular membrane are the most popular paths of intercellular mitochondrial transport [53]. In addition, Sansone et al., proposed the horizontal transfer of mitochondrial DNA from small EVs, isolated using sequential centrifugation, in breast cancer. The authors demonstrated that in this way, cancer-associated fibroblasts induced the escape from metabolic quiescence in hormonal therapy-sensitive or metabolically-dormant populations of breast cancer cells [54]. In line with these results, we found that small EVs released by breast cancer cells grown in the presence of leptin were enriched in mitochondrial elements such as “Mitochondrial inner membrane”, “Mitochondrial matrix”, “Mitochondrial protein complex”, “Mitochondrial membrane part”, “Outer membrane”, and “Respiratory chain”. In functional studies, using equal quantities of vesicles, Lep-EVs were able in an autocrine manner to modulate the metabolism of recipient breast cancer cells, by increasing the production of ATP, a major driver of aggressive cancer cell phenotypes [55], and favoring the energy production by the mitochondrial phosphorylation. Thus, we can speculate that the obesity hormone leptin sustains metabolism in breast cancer cells either directly, as previous reported [38,40], or indirectly, inducing the release from breast cancer cells of a large amount of small EVs [12] bearing a specific protein cargo of mitochondrial components. Moreover, our results also propose Lep-EVs as important contributors to the metabolic crosstalk within the immune breast cancer microenvironment promoting immune tolerance. Macrophages are the most abundant innate immune cells in the tumor microenvironment characterized by high plasticity in phenotype and function [56]. Classically, the tumor-associated macrophages (TAMs) are described to have an M2-like polarization state in which cells can exert pro-tumoral activities and induce the immune tolerance, although characteristics of M1-like anti-tumor macrophages have been also described [56], since a broad range of macrophage phenotypes exists. From a metabolic point of view, M1-like cells preferentially depend on glycolysis for energy production to give rise to a pro-inflammatory phenotype, whereas M2-like cells mainly obtain the ATP from tricarboxylic acid cycle and oxidative phosphorylation [57,58]. Interestingly, we found that Lep-EVs have the ability to influence the metabolic profile of the M0-macrophages toward the M2-like TAMs characterized by an increased oxygen consumption rate and an increased expression of the typical M2 markers as arginase 1, *IL-10*, *VEGF* and PD-L1. Functionally, macrophages polarized in the presence of Lep-EVs displayed a reduced phagocytosis, a process crucial to the innate immune response used by classically activated macrophages to engulf tumor cells. This could represent an additional mechanism by which breast cancer cells can reprogram adjacent immune cells to optimize the cancer cell pro-tumoral environment. 

Overall, we demonstrate in ERα-positive MCF-7 cells, a model of Luminal A subtype of breast cancer that the Lep-EVs were able to convey protein machinery required for mitochondrial metabolism with important impact on the metabolic behavior of recipient tumor and immune cells that can be located in situ or far from the site of release (e.g., the premetastatic niche). Since it is now accepted that targeting mechanisms sustaining mitochondria function hold great potential as an anticancer strategy with high therapeutic opportunities, these results provide new metabolic vulnerabilities that can be targeted for treating obesity-associated breast cancers.

## 4. Materials and Methods

### 4.1. Antibodies and Reagents

Anti-Tsg101 (sc-7964) and anti-Calnexin (sc-11397) were acquired from Santa Cruz Biotechnology (Dallas, TX, USA); anti-ALIX (EPR14314) was acquired from Abcam (Cambridge, UK). Leptin and Exosome-depleted FBS were obtained from Life Technologies (Monza, MB, Italy). Phorbol 12-myristate 13-acetate (PMA) was purchased from Merck (Burlington, MA, USA). 

### 4.2. Cell Cultures

Human MCF-7 breast cancer epithelial cells and Human THP-1 monocytic cell line (American-Type-Culture-Collection, Manassas, Virginia, USA) were stored and authenticated according to supplier’s instructions. MCF-7 cells were cultured in DMEM medium (Life Technologies) containing 10% FBS, 1% L-glutamine, 1% Eagle’s nonessential amino acids, and 1% penicillin-streptomycin (Life Technologies) at 37 °C with 5% CO2 air. THP-1 cells were cultured in RPMI-1640 medium (Life Technologies), supplemented with 10% FBS and 1% penicillin-streptomycin at 37°C in a humidified 5% CO_2_ air. Every six months, cells were authenticated by single tandem repeat analysis at our Sequencing Core; morphology, doubling times, estrogen sensitivity, and mycoplasma negativity were tested (MycoAlert, Lonza, Basel, Switzerland).

### 4.3. Isolation of Extracellular Vesicles

Cells were seeded at a density of 35 × 10^5^ cells/75 cm^2^ flask in 10 mL of growth medium and then incubated for 48 h in medium containing 10% Exosome-depleted FBS in the presence of vehicle or 500 ng/mL of leptin, and the isolated EVs have been named C-EVs and Lep-EVs, respectively. Briefly, at least five flasks/conditions were used. The conditioned medium was harvested, and extracellular vesicles (EVs) were isolated by differential ultracentrifugation method as previously described [12]. The EV pellet was resuspended in PBS and stored at −80 °C until use.

### 4.4. Transmission Electron Microscopy (TEM)

Whole EV extracts were fixed in 2% glutaraldehyde and absorbed onto formovar-coated grids for 20 min in a dry environment. The grids were examined in a Jeol JEM 1400 Plus electron microscope (JEOL USA, Inc., Peabody, MA, USA) at 80 kV.

### 4.5. Nanoparticle Tracking Analysis (NTA)

The size distribution and the concentration of particles were analyzed using NanoSight NS300 technology (Malvern Panalytical Ltd., Malvern, UK) and the assays were performed according to the recommendation of the instrument’s manufacturer as previously described [59].

### 4.6. Immunoblot Analysis

Cells and EVs were lysed in RIPA Buffer (50 mM Tris-HCl, 150 mM NaCl, 1% Nonidet P-40, 0.5% sodium deoxycholate, 2 mM sodium fluoride, 2 mM EDTA, and 0.1% SDS) containing a mixture of protease inhibitors (aprotinin, phenylmethylsulfonyl fluoride, and sodium orthovanadate). Equal amounts of cell and EV extracts were resolved on 11% SDS-polyacrylamide gel, as described [60]. The images were acquired using Odissey FC (Licor, Lincoln, NE, USA). The uncropped blots have been reported in Figure S3.

### 4.7. Proteomic Analysis

#### 4.7.1. Protein Digestion

50 μL of C-EVs and Lep-EVs were dissolved in 4% SDS buffer containing 100 mM Tris pH 8.0. To achieve complete lysis, samples were sonicated for three times. Then, proteins were reduced with the addition of 10 μL of DTT 500 mM and denaturated at 95 °C for 10′. Protein digestion was performed by the Filter-Aided Sample Preparation (FASP) method, as described in Wisniewski et al. [61], using the filter membrane Microcon (10 kDa, Millipore) [61,62]. 60μL of 50 mM TEAB buffer and 200 ng of trypsin “proteomics grade” (Merk Life Science, Milano, Italy ) were added to each sample for digestion at 37 °C overnight. 

#### 4.7.2. Protein Purification

The following day, 140 µL of H_2_O was added and the samples were centrifuged at 14,000× *g* for 20 min to collect 200µL of digest0 μL of each sample were then purified by StageTip SCX as reported in Rappsilber et al. [63]. The digested samples were acidified by adding 80% acetonitrile-0.5% formic acid (Wash B, 200μL) and loaded into pipette tip with a layer of SCX resin (Millipore extraction disks) previously conditioned with Wash A (0.5% FA and 20% ACN). After two washes with Wash B and A peptides have been eluted with 10 μL of 500 mM ammonium acetate (AA) and 20% of ACN. All peptide fractions were fully dried and reconstituted in 10 μL of 2% ACN/0.1% FA (*v*/*v*). One μL of purified digest have been used for Nano Liquid Cromatography-Tandem Mass Spectometry (Nano-LC-MS/MS).

#### 4.7.3. Mass Spectometry Analysis 

LFQ (label-free quantification) analysis was performed with an LC-MS/MS system consisting of an EASY-LC-1000 chromatograph and a Q-Exactive mass spectrometer (Thermo Fisher, Waltham, MA, USA). Complex peptide mixtures were separated using analytical column with the following specifications: length, 14 cm; inner diameter, 75 μm; 3 μm-C18 silica particles. All the LC analyses were carried out at 0.3 μL/min flowrate and peptides were eluted using two mobile phases: A, consisting of 2% ACN, 0.1% FA (v/v); B, consisting of 80% ACN, and 0.1% FA (*v*/*v*) with the following gradient: from 0% mobile phase B to 7% mobile phase B in 1 sec, 7–40% B in 30 min, 48%–100% B in 8 min, 100% B in 5 min, and back to the initial conditions (0% B) in 2 min. LC-MS/MS analysis were performed in data-dependent acquisition (DDA). The full scan *m*/*z* range was 350–1800, followed by MS/MS scans on the 12 most intense precursor ions. DDA analysis was performed with resolution for full MS scan of 70,000 and of 35,000 for MS/MS scan; the isolation window was 1.6 *m*/*z*. The maximum injection time was set to 50 ms for full MS scans and to 120 ms for MS/MS scans.

#### 4.7.4. Data Analysis

Raw MS data were analyzed using MaxQuant software using the Andromeda search engine. The MS/MS spectra were searched against a Homo sapiens reference proteome. For LF samples the following settings were used: fixed modifications: carbamidomethyl (C); variable modification: oxidized methionine (M) and acetyl (N-terminus). Unique peptides (minimum 1 peptide per protein group) were used for protein identification. The Match-between-runs (MBR) option was activated. For statistical analysis of MaxQuant output, Perseus software was used as follows: the LFQ intensity of proteins from the MaxQuant analysis were imported and contaminants, reverse identification, and proteins only identified by site were excluded from further data analysis. Data were transformed in logarithmic scale (log2). After filtering (at least four valid LFQ values in at least one group), remaining missing LFQ values were imputed from a normal distribution (width, 0.3; down shift, 1.8). Finally, for all the data sets, two-sample t test was used to assess statistical significance of protein abundances. A scaling factor was used for correction: s0: 0.2. The mass spectrometry proteomics data have been deposited to the ProteomeXchange Consortium via the PRIDE partner repository with the dataset identifier PXD037266.

### 4.8. Analysis of Extracellular Vesicles Proteome Cargoes

We used the Bioinformatics & Evolutionary Genomics (https://bioinformatics.psb.ugent.be/webtools/Venn/, accessed on 1 November 2021) tool to construct a Venn diagram to evaluate the potential overlapping between our list proteins identified in the EVs and EV proteins previously annotated in the Vesiclepedia (http://microvesicles.org, accessed on 1 November 2021) and Exocarta databases (http://www.exocarta.org/, accessed on 1 November 2021).

### 4.9. Functional Enrichment and Biological Network Analysis

Gene set enrichment analysis (GSEA) was performed for better investigating the function of differentially up-regulated proteins detected in Lep-EVs compared to C-EVs. In particular, selected proteins were annotated and analyzed according to the three organizing principles of Gene Ontology (BP: Biological Process, MF: Molecular Function, CC: Cellular Component) using the GSEA^®^ analysis software (https://www.gsea-msigdb.org/gsea/index.jsp, accessed on 12 July 2021). Normalized enrichment scores (NES) >1 and Benjamini-Hochberg adjusted *p* values < 0.05 were set as the thresholds to filter out significant terms.

### 4.10. Quantitation of Cellular ATP Levels

Intracellular ATP levels were assessed using an ATP Assay Kit (ab83355, Abcam) and following the manufacturer’s protocol. Briefly, 35 × 10^4^ MCF-7 cells were plated in Exo-depleted medium for 24 h, then cells were treated or not with an equal amount (2μg/mL) of C-EVs or Lep-EVs for a further 48 h. Optical density (570 nm) was measured using microplate reader (MultiskanTM SkyHigh, Life Technologies). 

### 4.11. Evaluation of Mitochondrial Mass and Mitochondrial Membrane Potential

MitoTracker^®^ Deep Red (mitochondrial mass evaluation) or MitoTracker^®^ Orange CM-H2TMRos (mitochondrial membrane potential evaluation) (Life Technologies) were used to evaluate mitochondrial mass and membrane potential, respectively, as previously reported in Nigro et al. [64]. Briefly, 35 × 10^4^ cells were plated in full media for 24 h, then cells were treated or not with 2 μg/mL of C-EVs or Lep-EVs for 48 h. Next, cells were trypisinized, collected, and incubated with a MitoTracker staining solution (10 nM in PBS) for 30–60 min at 37 °C. Cells were then harvested, re-suspended in PBS and, analyzed by flow cytometry (CytoFLEX Beckman, Beckman Coulter, Milan, Italy). Data analysis was performed using CytExpert Beckman Coulter software (Beckman Coulter Milan, Italy).

### 4.12. Seahorse XFe96 Metabolic Profile Analysis

Mito Stress test and Glycolysis Stress test were employed to assess the cellular metabolic profile in terms of real-time oxygen consumption rates (OCR) and extracellular acidification rates (ECAR) using the Seahorse Extracellular Flux (XFe-96) analyzer (Seahorse Bioscience, Agilent Technologies, North Billerica, MA, USA). Briefly, 10 × 10^3^ MCF-7 cells/well were seeded in a final volume of 150 μL into XFe-96 well cell culture plates for 24 h. The next day, cells were treated or not with 2 μg/mL of C-EVs, Lep-EVs or 500 ng/mL Lep for 48 h. After a wash in pre-warmed XF assay media (10 mM glucose, 1 mM Pyruvate, 2 mM L-glutamine for OCR measurements; 2 mM L-glutamine for ECAR measurements, and adjusted at 7.4 pH) cells were maintained at 37 °C, in a non-CO_2_ incubator for 1 h0 μM Oligomycin (able to inhibit ATP synthase), 9 μM FCCP (that uncouples oxygen consumption from ATP production) and 10 μM Rotenone/10 μM Antimycin A mix (that specifically target complex I and complex III of electron transport chain, respectively) were sequentially injected to calculate cellular respiration parameters. Instead, glycolytic parameters calculation was obtained by sequential injections of 80 mM glucose (a glycolysis substrate), 9 μM Oligomycin (that through the inhibition of ATP synthase switches cell metabolism towards glycolysis) and 1 μM M2-deoxyglucose (2-DG) (a glucose analogue that competitively inhibits the glycolytic process). To study macrophage metabolism, 18 × 10^3^ THP-1 cells were seeded in XFe-96 96-wells, then PMA 100 nM was added for 24 h, to obtain M0 macrophages and this was followed by 1 day of rest in medium without PMA. Then, M0 macrophages were treated or not with 2 μg/mL of C-EVs or Lep-EVs for 48 h. Mito Stress and Glycolysis Stress tests were assessed as reported above. All experiments were performed three times independently in quintuplicate. Data were normalized to cell mass determined using the Sulforhodamine B (SRB) assay.

### 4.13. Sulforhodamine B (SRB) Assay

After Seahorse analysis, cells were washed, fixed, and stained with the SRB dye (Acid Red 52) (Sigma Aldrich). Briefly, cells were fixed with 10% trichloroacetic acid (TCA) for 1 h in cold room, and then incubated with SRB for 30 min, washed twice with 1% acetic acid, and air dried for at least 3 h. Finally, the incorporated dye was then released from the cells with 10 mM Tris pH 8.8 solution and the signal was detected spectrophotometrically at a wavelength of 565, (MultiskanTM SkyHigh, Life Technologies). 

### 4.14. Total RNA Extraction and Reverse Transcription Real-Time PCR Assay

50 × 10^4^ THP-1 cells/well were plated in 6-multiwell, then PMA 100 nM was added for 24 h. After 24 h of rest, M0 macrophages were treated or not with 2 μg/mL of C-EVs or Lep-EVs for 48 h. Total RNA was extracted from cells using TRIzol reagent (Life Technologies) and evaluation of gene expression was performed by real-time reverse transcription PCR using a RETROscript kit (Life Technologies). The cDNAs obtained were diluted 1:10 in nuclease-free water and 5 µL were analyzed in triplicates by real-time PCR in an iCycler iQ Detection System (Bio-Rad, Hercules, CA, USA) using SYBR Green Universal PCR Master Mix with 1 mmoL/L of each primer in a total volume of 20 µL reaction mixture following the manufacturer’s recommendations. Negative control contained water instead of first strand cDNA was used. Primers used were listed in Supplementary Table S1. 

Samples were normalized on 18S rRNA content. Relative gene expression levels were calculated as reported [65].

### 4.15. Phagocytosis Assay

Phagocytic activity of macrophages were assessed using the Phagocytosis Assay Kit (Cayman Chemical, Ann Arbor, MI, USA) as previously performed [30]. Phagocytosis was evaluated by counting at least 150 cells for each experimental condition and calculated as the percentage of macrophages that engulfed fluorescein-labeled rabbit IgG-coated latex beads with respect to total macrophages.

### 4.16. Flow Cytometry 

THP-1 cells were seeded in 60 mm dishes, differentiated as previously described and untreated or treated with C-EV or Lep-EV for 48 h before staining. Cells were washed with cold PBS; detached with versene and centrifuged, and the obtained pellet was resuspended in a total of 100 µL of cold PBS containing 5 µL of PE anti-CD80 antibody (# 557227; Becton Dickinson, MI, Italy) or FITC anti-CD206 antibody (# 321103; BioLegend, San Diego, CA, USA). After incubation (30 min at 4 °C), cells were washed with 1× PBS and centrifuged at 500× *g* for 5 min and then re-suspended in of 1× PBS and analyzed by flow cytometry (CytoFLEX Beckman, Beckman Coulter, Milan, Italy). Data analysis was performed using CytExpert Beckman Coulter software (Beckman Coulter, Milan, Italy). Isotype matched negative control antibodies were used as negative control sample.

### 4.17. Statistical Analysis

Statistical analysis was performed using Student’s *t* test using GraphPad Prism 8 (GraphPad Software, Inc., San Diego, CA, USA) and reported as the mean ± SD or ± SEM, as indicated, of at least three independent experiments, each performed in triplicate. 

## Data Availability

The data presented in this study are available on request from the corresponding author. The mass spectrometry proteomics data have been deposited to the ProteomeXchange Consortium via the PRIDE partner repository with the dataset identifier PXD037266.

## Supplementary Materials

The following supporting information can be downloaded at: https://www.mdpi.com/article/10.3390/ijms232112941/s1.
