# Supplementary material for: Proteomic Profiling of Extracellular Vesicles Released by Leptin-Treated Breast Cancer Cells: A Potential Role in Cancer Metabolism
Source: Int J Mol Sci. 2022 Oct 26;23(21):12941. doi: 10.3390/ijms232112941 (PMC9659287; doi:10.3390/ijms232112941)
Supplement: Supplementary file 1 [file ijms-23-12941-s001.zip › Table S1.pdf]

Table S1. Oligonucleotide sequences used in this study.

Gene Name	Gene Symbol	Primer Sequence
Interleukin 6	*IL-6*	Forward: 5′- AACCTGAACCTTCCAAAGATGG -3′<br>Reverse: 5′- TCTGGCTTGTTCCTCACTACT-3′
Interleukin 1 beta	*IL-1β*	Forward: 5′- CACGATGCACCTGTACGATCA-3′<br>Reverse: 5′- GTTGCTCCATATCCTGTCCCT-3′
Tumor Necrosis Factor alpha	*TNF-α*	Forward: 5′- ATGAGCACTGAAAGCATGATCC-3′<br>Reverse: 5′- GAGGGCTGATTAGAGAGAGGTC-3′
CD163 molecule	*CD163*	Forward: 5′- ACTTGAAGACTCTGGATCTGCT-3′<br>Reverse: 5′- CTGGTGACAAAACAGGCACTG-3′
Interleukin 10	*IL-10*	Forward: 5′- ACTTTAAGGGTTACCTGGGTTGC-3′<br>Reverse: 5′- TCACATGCGCCTTGATGTCTG -3′
Interleukin 1Ra	*IL-1Ra*	Forward: 5′- CATTGAGCCTCATGCTCTGTT-3′<br>Reverse: 5′- CGCTGTCTGAGCGGATGAA -3′
Vascular Endothelial Growth Factor	*VEGF*	Forward: 5'-GAGATGAGCT TCCTACAGCAC-3′<br>Reverse: 5'-TCACCGCCTCGGCTTGTCACAT-3′
Cluster of differentiation 274	*CD274*	Forward: 5'-GGCATTTGCTGAACGCATT-3′<br>Reverse: 5'- ACAATTAGTGCAGCCAGGT -3′′
Arginase-1	*ARG1*	Forward: 5'-GGAGTCATCTGGGTGGATG-3′<br>Reverse: 5'- GGCACATCGGGAATCTTTCCT -3′′
18S Ribosomal RNAs	*RNA18S*	Forward: 5′-CGGCGACGACCCATTCGAAC-3′<br>Reverse: 5′-GAATCGGAACCCTGATTCCCCGTC-3′
