# Supplementary figures and images for: Proteomic Profiling of Extracellular Vesicles Released by Leptin-Treated Breast Cancer Cells: A Potential Role in Cancer Metabolism
Source: Int J Mol Sci. 2022 Oct 26;23(21):12941. doi: 10.3390/ijms232112941 (PMC9659287; doi:10.3390/ijms232112941)

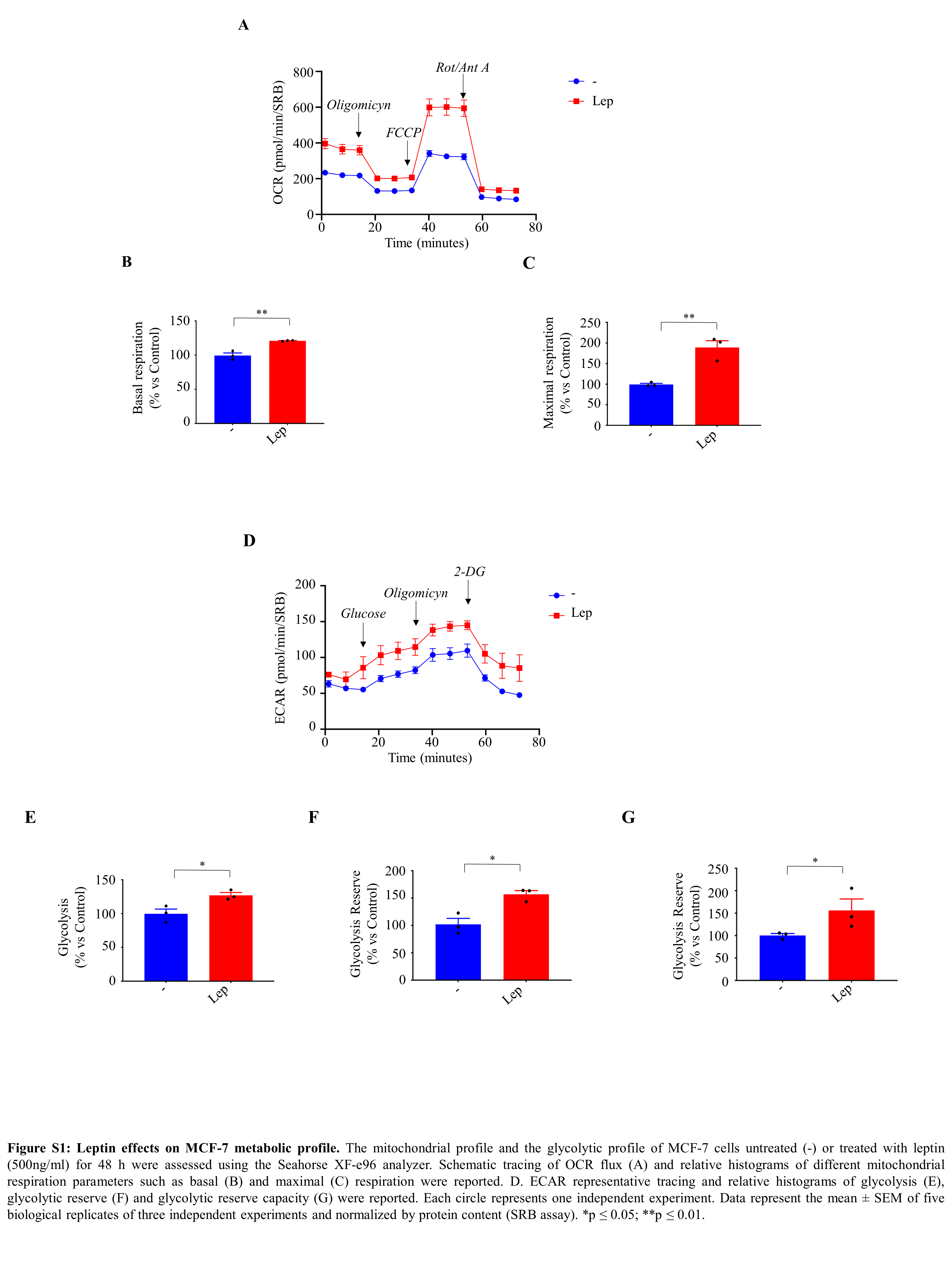

Supplement: Supplementary file 1 [file ijms-23-12941-s001.zip › Figure S1.tif]

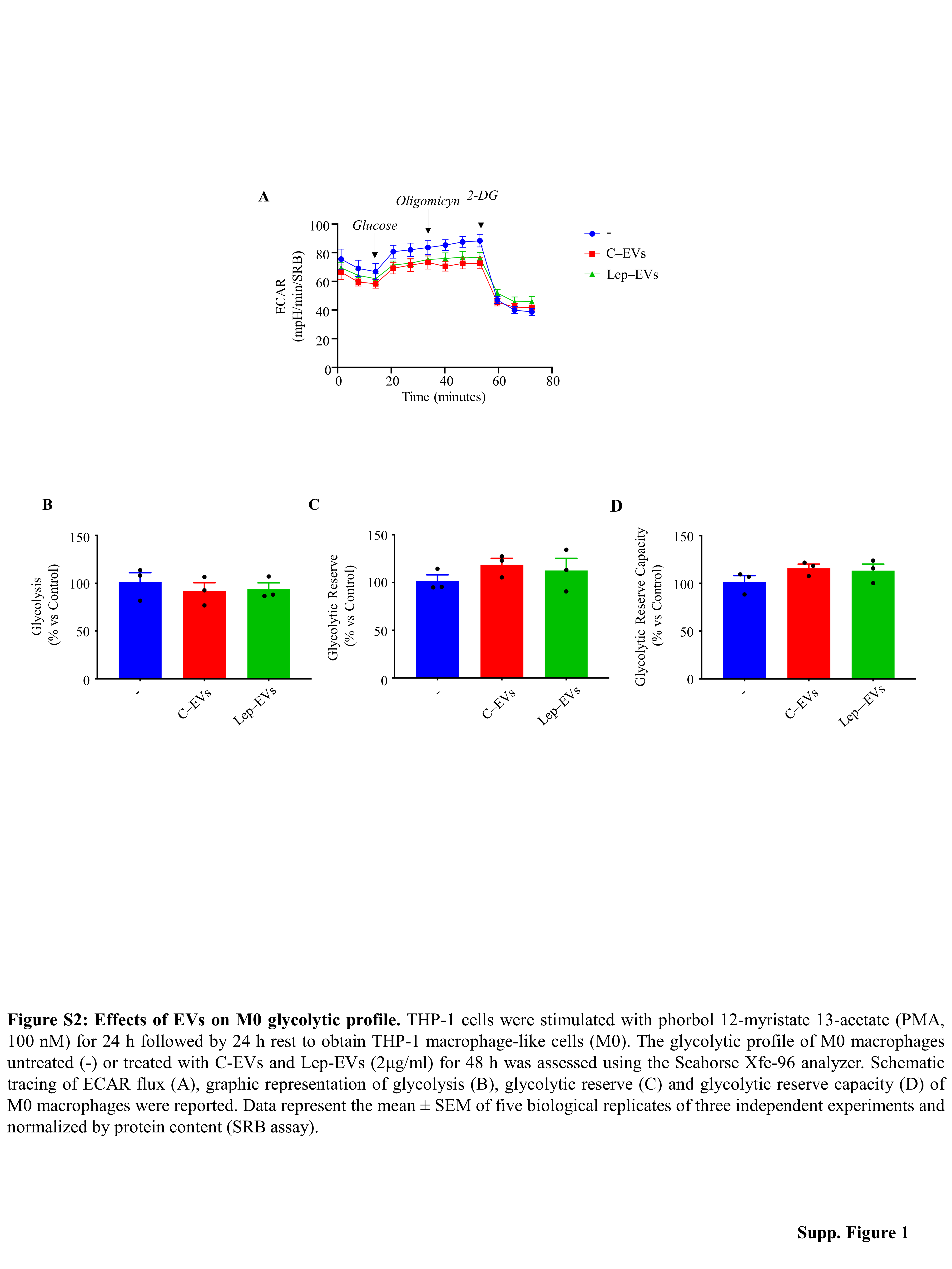

Supplement: Supplementary file 1 [file ijms-23-12941-s001.zip › Figure S2.tif]

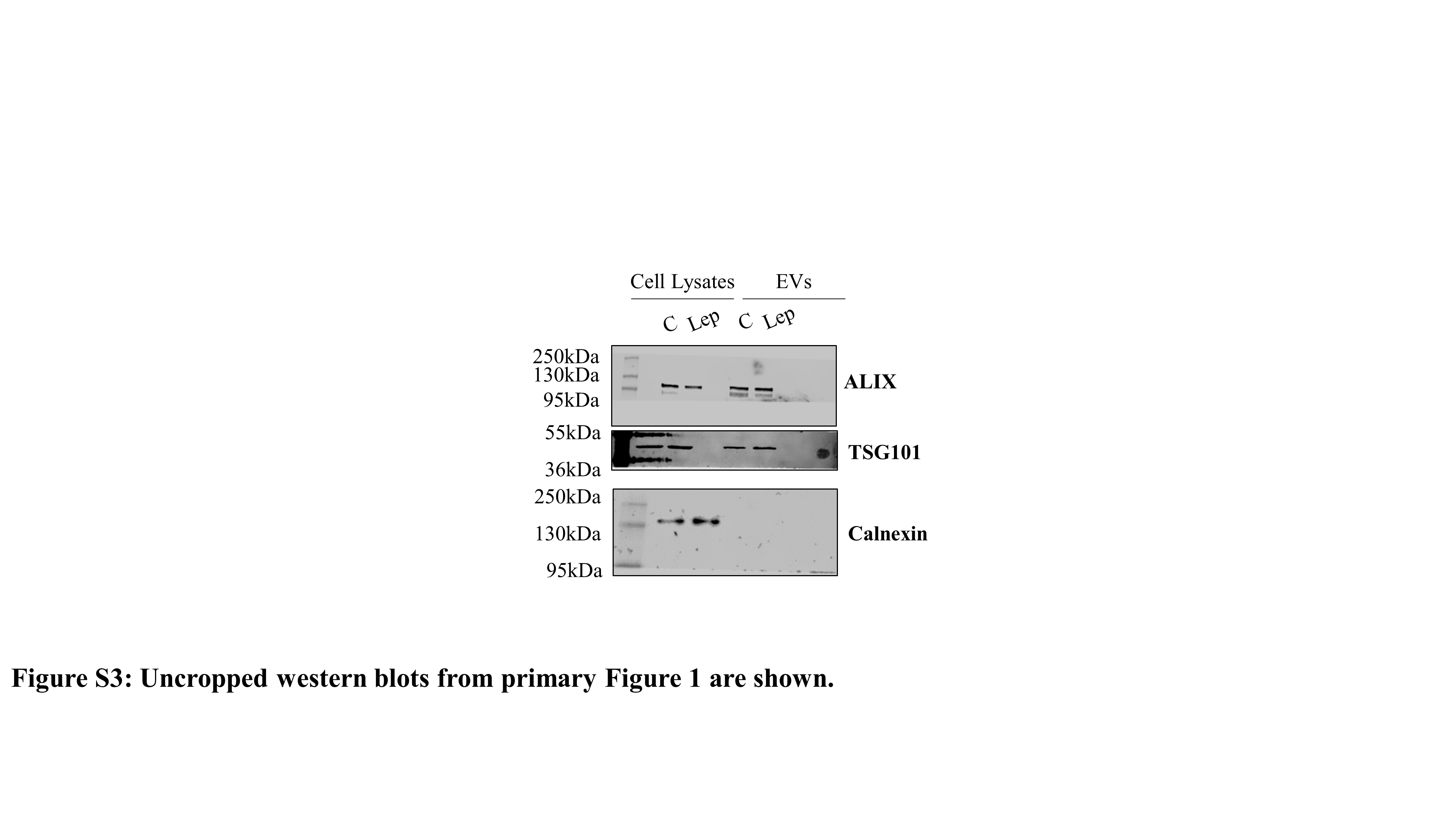

Supplement: Supplementary file 1 [file ijms-23-12941-s001.zip › Figure S3.tif]
